# Detection of Low-Level Mixed-Population Drug Resistance in *Mycobacterium tuberculosis* Using High Fidelity Amplicon Sequencing

**DOI:** 10.1371/journal.pone.0126626

**Published:** 2015-05-13

**Authors:** Rebecca E. Colman, James M. Schupp, Nathan D. Hicks, David E. Smith, Jordan L. Buchhagen, Faramarz Valafar, Valeriu Crudu, Elena Romancenco, Ecaterina Noroc, Lynn Jackson, Donald G. Catanzaro, Timothy C. Rodwell, Antonino Catanzaro, Paul Keim, David M. Engelthaler

**Affiliations:** 1 Translational Genomics Research Institute, Flagstaff, AZ, United States of America; 2 San Diego State University, San Diego, CA, United States of America; 3 Phthisiopneumology Institute (PPI), Chisinau, Republic of Moldova; 4 University of California San Diego, San Diego, CA, United States of America; 5 University of Arkansas College of Education and Health Professions, Fayetteville, AR, United States of America; 6 Center for Microbial Genetics & Genomics, Northern Arizona University, Flagstaff, AZ, United States of America; University of California, San Francisco, UNITED STATES

## Abstract

Undetected and untreated, low-levels of drug resistant (DR) subpopulations in clinical *Mycobacterium tuberculosis* (*Mtb*) infections may lead to development of DR-tuberculosis, potentially resulting in treatment failure. Current phenotypic DR susceptibility testing has a theoretical potential for 1% sensitivity, is not quantitative, and requires several weeks to complete. The use of “single molecule-overlapping reads” (SMOR) analysis with next generation DNA sequencing for determination of ultra-rare target alleles in complex mixtures provides increased sensitivity over standard DNA sequencing. Ligation free amplicon sequencing with SMOR analysis enables the detection of resistant allele subpopulations at ≥0.1% of the total *Mtb* population in near real-time analysis. We describe the method using standardized mixtures of DNA from resistant and susceptible *Mtb* isolates and the assay’s performance for detecting ultra-rare DR subpopulations in DNA extracted directly from clinical sputum samples. SMOR analysis enables rapid near real-time detection and tracking of previously undetectable DR sub-populations in clinical samples allowing for the evaluation of the clinical relevance of low-level DR subpopulations. This will provide insights into interventions aimed at suppressing minor DR subpopulations before they become clinically significant.

## Introduction

The incidence of multidrug-resistant tuberculosis (MDR-TB) is on the rise; from 2011–2012 the World Health Organization (WHO) reported a 42% increase in detected MDR-TB cases and predicted that the number of cases will likely rise, as only 25% of new MDR-TB cases are actually diagnosed[1]. A major hurdle in the detection of MDR-TB is the length of time required to obtain a drug resistance profile. Traditionally, diagnosing drug resistant TB (DR-TB) in clinical samples requires phenotypic drug susceptibility testing (DST). *Mycobacterium tuberculosis* is a slow growing organism, and thus phenotypic DST takes weeks to months to complete. During this time, patients may not be under appropriate therapy and are both a continued MDR transmission and high mortality risk. There is a critical need for fast and reliable determination of drug resistance profiles to inform patient treatment[1].

The identification of clinically relevant genetic marker loci has led to the development of effective and rapid molecular diagnostics for DR-TB[2–4]. A number of DR genetic loci have been well characterized in *M*. *tuberculosis*, accounting for 90–98% of the observed phenotypic resistance to a number of anti-tuberculosis drugs[2]. These loci are the basis for a number of assays that have been implemented for rapid resistance detection. There are several molecular approaches to detect drug resistance in *M*. *tuberculosis*: restriction fragment length polymorphism[5], line probe[6–8], molecular beacon[9], Xpert MTB/RIF assay[10], digital PCR[11], single-strand-conformation-polymorphism (PCR-SSCP)[12, 13], real-time PCR [14, 15], sanger sequencing [16], whole genome sequencing [17] and pyrosequencing[18]. These approaches are limited, however, by either suboptimal resistant allele detection levels for mixtures, by needing pure isolate DNA, or by the fact that they can only examine a few genomic targets at a time.

Mixtures of resistant subpopulations of *M*. *tuberculosis* within a larger population of susceptible pathogens have been shown to be present in up to 30% of tuberculosis (TB) patients[6, 19], and is undoubtedly more frequent at currently undetectable levels. The presence of resistant subpopulations in an individual patient’s infection creates difficulties in the interpretation of rapid molecular drug resistance tests because it leads to ‘indeterminate’ test results[6, 20] and it may lead to the development of DR-tuberculosis[5]. Current technologies are limited to detecting resistant genotypes making up >10% of a population (pyrosequencing)[21], 5% (line probe assay)[22], and 1% of a mixed population (Mycobacteria growth indicator tube, MGIT)[22], furthermore, neither line probe or MGIT are able to accurately quantify the proportion of resistant subpopulations. This lack of sensitive subpopulation detection technology has prevented a comprehensive investigation of the importance of low-level (<1%) resistance mixtures in delayed sterilization of patient infections, acquired drug resistance (ADR) and treatment failure. Understanding the impact of lower level resistant subpopulations may help explain some of the inconsistencies observed in molecular and phenotypic diagnostics, TB culture conversion rates and treatment outcomes.

Massively parallel or next-generation sequencing (NGS) technology has altered the course of infectious disease detection and characterization. Unlike conventional sequencing techniques, NGS digitally tabulates the sequence of millions of DNA fragments from a pathogen target, thus allowing detection and quantification of minor genetic components within a heterogeneous mixture[23]. While the hurdle of data analysis is being overcome for fine scale subpopulation characterization, the error rates associated with the NGS sequencing platforms are still a significant challenge. The intrinsic error rate of the sequencing process and the depth of sequence coverage set the current detection limits of rare variants or minor subpopulations. In order to increase the sensitivity of minor subpopulation detection with NGS a number of different methods of “data cleaning”[24–26] and other bioinformatics techniques[27–29] have been developed to effectively lower the sequencing error rate. Recently, library preparation modifications have been designed to lower the sequencing error rate and thus improve the limit of detection of a minor subpopulation[23, 30]. However, these techniques are still intrinsically tied to the sequencing process error rate or reduce effective coverage drastically.

Single Molecule Overlapping Read (SMOR) analysis involves designing NGS amplicon libraries in such a way that allows for the complete overlap of forward and reverse paired-end reads from the same DNA molecule. This provides two independent base calls at each position within the same DNA fragment, which significantly lowers the probability of an erroneous base call, theoretically by orders of magnitude[31]. The use of amplicon sequencing of target regions of importance increases sequence coverage in a cost effective manner, and has been used in other population diversity research[32, 33]. Coupling the vastly increased depth of coverage attainable with amplicons on NGS platforms with overlapping reads, will significantly lower the effective limits of detection of minor subpopulations.

Here we present the use of SMOR analysis to simultaneously characterize 30 known drug-resistance conferring SNP loci contained within six *M*. *tuberculosis* gene regions for ultra-rare resistant subpopulation detection[2]. This method was evaluated and validated using *in vitro* mixtures of extensively resistant and pan-susceptible strains to detect and quantify resistant subpopulations down to 0.1%. Once validated, we used the SMOR method to evaluate mixed resistant/susceptible subpopulations in clinical *M*. *tuberculosis* samples. This targeted ligation free amplicon NGS approach can rapidly and comprehensively characterize the genetic DR profiles of a large number of patients’ infections at low cost, which will enable the detection and monitoring of drug resistant subpopulations over the course of treatment. The ability of NGS with SMOR analysis to detect and quantify ultra-rare antibiotic resistant subpopulations of *M*. *tuberculosis* is demonstrated, which will enable earlier detection of developing resistance during treatment of active disease.

## Materials and Methods

### DNA and Clinical Samples

For development and validation analyses, DNA was extracted from cultures of two strains housed in UCSD’s repository[34], one pan susceptible (strain 2–0112) and the other extensively drug resistant (strain 4–0009) (provided by University California, San Diego). 2–0112 and 4–0009 were cultured from single colony isolation and extracted using the QIAGEN Genomic-tip with Genomic DNA Buffer Set. The manufacturer's sample prep for bacteria was followed with modification to the lysis step. A bacterial lawn cultured on Middlebrook 7H11 plates was harvested by scraping with a sterile loop directly into Qiagen buffer B1-RNase and made homogenous by vigorous vortex mixing. This bacterial suspension was inactivated by incubation for 60 minutes in a water bath at 80°C. Upon cooling, lysozyme was added per the manufacturer recommendation and the suspension incubated at 37°C for 30 minutes. Proteinase K was added and the incubation at 37°C continued for an additional 60 minutes. The recommended volume of Qiagen Buffer B2 was added and the suspension incubated at 50°C overnight (up to 16 hours). Following lysis the Genomic-tip protocol in the Qiagen Genomic DNA Handbook was followed.

For clinical validations, DNA from twelve sputum samples from separate TB patients in Moldova were provided[35]. The Moldova *M*. *tuberculosis* DNA from these clinical samples was extracted directly from pooled sputum samples, which have been decontaminated and concentrated by the NALC-NaOH method. The pool is a combination of a spot sample collected on the day of enrollment and a sample produced first thing the next morning. Sputum DNA was extracted by GenoLyse (Hain lifescience) following manufactures recommendations. Human research conduct: The study was reviewed and approved by institutional review boards at UCSD (University Of California, San Diego Human Research Protections Program) and Moldova (Ethics Committee Of Phthisiopneumology Institute, Public Health Medical Institution). The study was also registered with ClinicalTrials.gov (#NCT02170441). Written informed consent was obtained from all participants. Study participation did not alter the standard of care.

### Phenotypic Drug Susceptibility Testing

Each clinical isolate was subjected to standardized DST to INH, RIF, moxifloxacin (MOX), ofloxacin (OFX), amikacin (AMK), kanamycin (KAN) and capreomycin (CAP) on the Mycobacterial Growth Indicator Tube (MGIT) 960 platform, with EpiCenter software using standard manufacturer protocols (BD Diagnostic Systems, Franklin Lakes, NJ, USA). First-line drugs were available from Becton Dickinson, the second-line drugs testing were performed using validated critical concentrations of in-house (locally prepared by Moldova) drug solutions compatible with the WHO recommendations: 2.0 μg/ml for OFX, 0.25 μg/ml for MOX, 1.0 μg/ml for AMK, and 2.0 μg/ml for CAP[36]. As there were no published WHO recommended critical concentrations for KAN DST by MGIT 960 at the time of the study, we used 2.5 μg/ml based on the literature[37, 38] (details in Hillery et al [35]).

### Universal Tail Amplicon Sequencing Targets

**Fig 1:**
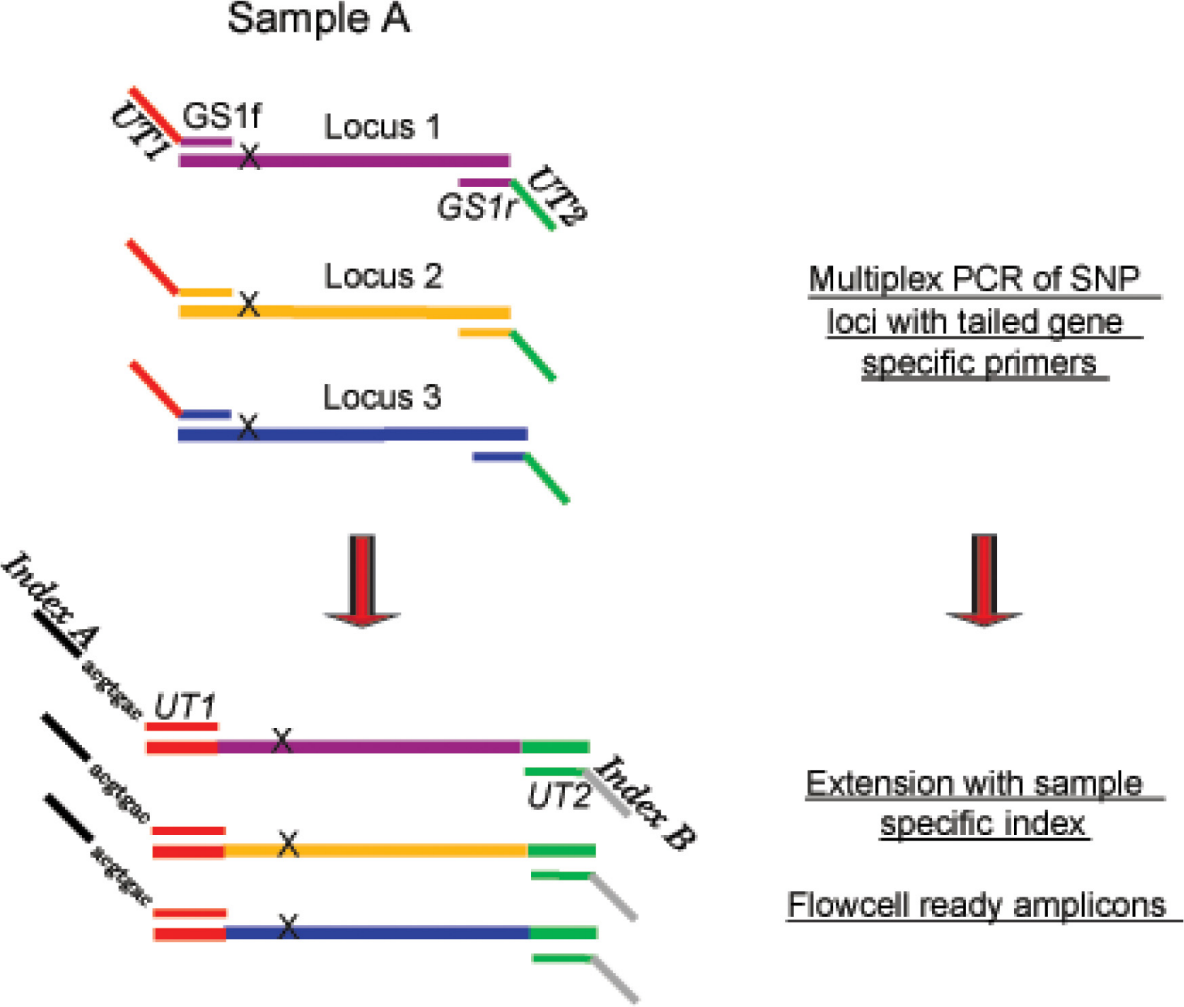
Illustration of universal tail approach. In a multiplex PCR, all *M*. *tuberculosis* gene specific targets are amplified with universal tailed primers. A second PCR extends the amplicon with a sample specific index that allows for several samples to be pooled together for sequencing. GS: gene specific primer; UT: universal tail sequence; Index A: Primer that includes the UT1 sequence and Illumina index sequence; Index. B: primer that includes the UT2 sequence and the Illumina sequence to bind to the flowcell.

Primer sets targeting 6 gene regions in *M*. *tuberculosis* containing all frequently detected drug resistance conferring SNPs were designed based on previously developed primers [2, 39–41] (S1 Table). The target amplicons were between 272–370 bp long. The size of the amplicons was dependent on the positions of the SNPs and the constraints of the length of the sequencing read. The target amplicons are comprised of 30 previously published SNP loci conferring resistance to INH, RIF, OFX, MOX, AMK, KAN, and CAP[2](S2 Table), six additional SNP loci were included that were of interest of possible resistance or phylogenetic use. For SMOR analysis each SNP position requires overlapping reads from one DNA molecule, thus coverage by both read 1 and read 2. Along with *M*. *tuberculosis* specific sequence, each primer has a universal tail sequence (red and bold sequences in S1 Table). All forward primers have one universal tail sequence while all the reverse primers have a second universal tail sequence. In a single Gene Specific multiplex PCR reaction, all six target amplicons are synthesized with the universal tail sequence added to the amplicons (Fig 1). The PCR parameters are as follows: initial denaturation at 98°C for 1 min, twenty-five cycles of denaturation at 98°C for 10 sec, annealing at 60°C for 15 sec, and extension at 72°C for 20 sec and a final extension at 72°C for 2 min. A single 25 uL PCR reaction contains 2 uL of DNA, 12.5 uL of Q5 Hot Start High-Fidelity 2X Master Mix (New England Biolabs Inc.), 4 uL of a primer mix, 1.5 uL of molecular grade H_2_O, and 5 uL of 5 M Betaine solution (Sigma-Aldrich). The primer mix contains the following final concentration of primers, where forward and reverse primer concentrations are equal for each target: 100 nM of *gyrA*, 400 nM *eis*, 100 nM *rpoB*, 200 nM *katG*, 400 nM *inhA*, and 400 nM *rrs*. After PCR, the reaction is cleaned up using a 1X Agencourt AMPure XP bead (Beckman Coulter) clean up with elution in 25 uL of a 10 mM Tris-HCl 0.05% Tween 20 solution.

### Index Extension—library creation

A second PCR adds a specific index sequence, based on the Kozarewa and Turner 8 bp indexing scheme[42], to the amplicons using the universal tail sequences on either end of the amplicon (S3 Table). At the end of the Index Extension PCR there is a sequencer ready amplicon library (Fig 1). The PCR parameters are as follows: initial denaturation at 98°C for 2 min, six cycles of denaturation at 98°C for 30 sec, annealing at 60°C for 20 sec, and extension at 72°C for 30 sec and a final extension at 72°C for 5 min. A single 50 uL PCR reaction contains 2 uL of template, 25 uL of 2x KAPA HiFi HotStart ReadyMix (KAPA biosystems), 2 uL of the 10 uM common universal tail primer, 2 uL of 10 uM specific index universal tail primer, 9 uL of molecular grade H_2_O, and 10 uL of 5 M Betaine solution (Sigma-Aldrich). After PCR the reaction was cleaned using a 0.8x Agencourt AMPure XP bead (Beckman Coulter) clean up with elution in 40 uL of a 10 mM Tris-HCl 0.05% Tween 20 solution to remove primer dimer. All completed libraries were run on the Bioanalyzer 2100 (Agilent Technologies) for confirmation of target amplification.

### Pooling and Sequencing

By adding sample specific index sequences to the amplicons, pools of several samples are made for sequencing. Each individual library, with six target amplicons, is quantified using KAPA Library Quantification Kit—Illumina/ABI Prism (KAPA biosystems) qPCR, and pooled in equal molar concentrations. Twenty-five individual libraries were pooled together for sequencing to target at least 100,000x coverage, allowing for extremely low-level detection of resistant alleles. At least 25% of each sequencing run was filled with whole genome or PhiX control samples to ensure base diversity and reduce complications with sequencing. For the validation a single sequencing pool was sequenced on the Illumina MiSeq platform using 2x300bp version 3 sequencing chemistry (Illumina). If PhiX control was not used for base diversity (e.g., at 25% of a sequencing run), then it was spiked in a low concentration (i.e., 1–5%) in each run for overall error rate examination. In all runs the pure susceptible isolate was run as a SMOR error control for the analysis. Novel Read 1, Read 2, and indexing sequencing primers were used for sequencing (S3 Table) and were added for a final concentration of 0.5 uM as per Illumina’s recommendation. All sequencing read files were deposited in NIH Short Read Archive (Bioproject # PRJNA271805).

### *In vitro* mixtures/ Multiplex validation

DNA from two strains of *M*. *tuberculosis*, one previously identified as pan susceptible (strain 2–0112) and the other identified as extensively drug resistant (strain 4–0009), were included in seven different mixtures. Each strain’s DNA was visualized on a 1% agarose gel, and a 16S real-time PCR assay[43] was used to normalize 2–0112 to 4–0009. Once normalized, the two strains were combined in the following approximate XDR to pan-susceptible mixtures: 70:30, 15:85, 10:90, 1:99, 0.1:99.9, 0.05:99.95, and 0.025:99.975. For each mixture, 1–5 ng was added to the Gene Specific multiplex PCR, which was run in five replicates per mixture, all replicates were sequenced to establish reproducibility of SMOR results. All 36 SNP loci were examined; however the seven different mixtures contain six known allelic differences in resistant conferring SNP loci. A dilution curve of the 10% XDR-TB mixture was run, starting with 5 ng and serial diluting down to 5 fg to find the lower limit of DNA input into the gene specific PCR steps. The *M*. *tuberculosis* specific primer sets were blasted against an extensive *Mtb* genomic database from PATRIC, and were screened across a panel of 93 isolates of 48 clinically relevant species (S4 Table) and three *M*. *tuberculosis*-negative human sputum samples for specificity analysis. Additionally, human DNA (Promega) was spiked into a 160 pg *M*. *tuberculosis* mixture to examine the effect of the presence of large amounts (2,000 fold difference based on mass) of background DNA on the multiplexed sample.

### Sequence data analysis

The reads generated for each mixture were trimmed *in silico* based on Illumina adapter and universal tail primer sequences using Trimmomatic[44] to ensure the removal of adapter sequence and the proper mapping of overlapping reads. The trimmed reads were mapped against amplicon specific reference sequences developed from *M*. *tuberculosis* H37Rv (accession no. NC_000962, NCBI), using Novoalign (Novocraft.com) and the default parameters. Both the trimming and the alignment tools take into account the quality scores from the sequencer, and thus reduce error rates in the overall alignment. The SMOR analysis script (https://github.com/TGenNorth/SMOR) automates the process of acquiring counts within an amplicon at each position of interest (i.e., SNP loci). The script is preloaded with a set of amplicon identifiers and position numbers identifying the positions of interest, prior to use. The script can be altered to contain additional positions within the amplicons, or remove positions as needed. Additionally more gene targets can be included in the analysis, as long as amplicon identifiers and position numbers are added to the script. The script accepts a '.bam' file as input, and searches this file for all paired reads that concurrently map to regions covering that position on that gene. When every relevant read pair is collected, a tally is made of the frequency at which each nucleotide appears at that position on both reads. This information is then presented to the user as a table of counts for each position of interest (S5 Table). For example, the script may report the detection of forty pairs of reads where both reads had a 'C' at the position of interest, and one pair of reads where both had a 'T'. For analysis purposes “erroneous alleles” were defined as the alternate bases (i.e., not the wild-type or known drug-resistant conferring mutations at each particular position). The user is also given a conventional count of nucleotides found at that position without “read pairing data” (referred to as “standard NGS analysis”). Additionally, the user is provided with a raw list of all paired data that includes cases where the reads in a pair disagree. Paired reads that disagree are considered sequencing errors because the only way for the reads from the same DNA molecule to disagree in a haplotype organism is due to sequencing error. For the SMOR analysis only counts of pairs that matched are used in the analysis, versus standard NGS analysis where pairing information is ignored.

### Clinical samples

Analysis of the DNA extracts from clinical samples was performed as described above for mixture analysis with the following additions. To ensure library concentrations were above the threshold for sequencing the volume of DNA was increased to 3.5 uL into the Gene Specific PCR and increased the number of cycles to 38. For four of the clinical samples (21–0017, 21–0045, 21–0067, and 22–0006) the Index Extension PCR was increased to 16 cycles, due to low levels of target DNA, as seen with the Bioanalyzer analysis (see “Index Extension – library creation”).

### Statistical Analysis

Analysis of increasing error rates due to the addition of PCR cycles was tested using a one-way ANOVA. The examination of the variation in resistant SNP allele frequencies with extremely low DNA input was preformed with a 2-sided F test of equal variance. Finally the analysis of how many organisms must be present in the Gene Specific PCR to target at least one organism of the resistant population being present was done using the Poisson distribution. All statistical analysis was conducted in JMP (version 10).

## Results

SMOR analysis was developed and validated with *in vitro* DNA mixtures of resistant and susceptible *M*. *tuberculosis* strains from clinical samples, and then applied to DNA extractions direct from clinical sputum samples to demonstrate the utility of the assay for detection of mixed resistant/susceptible SNP alleles in a clinical relevant time frame.

### Validation: *In vitro* Mixtures

**Fig 2:**
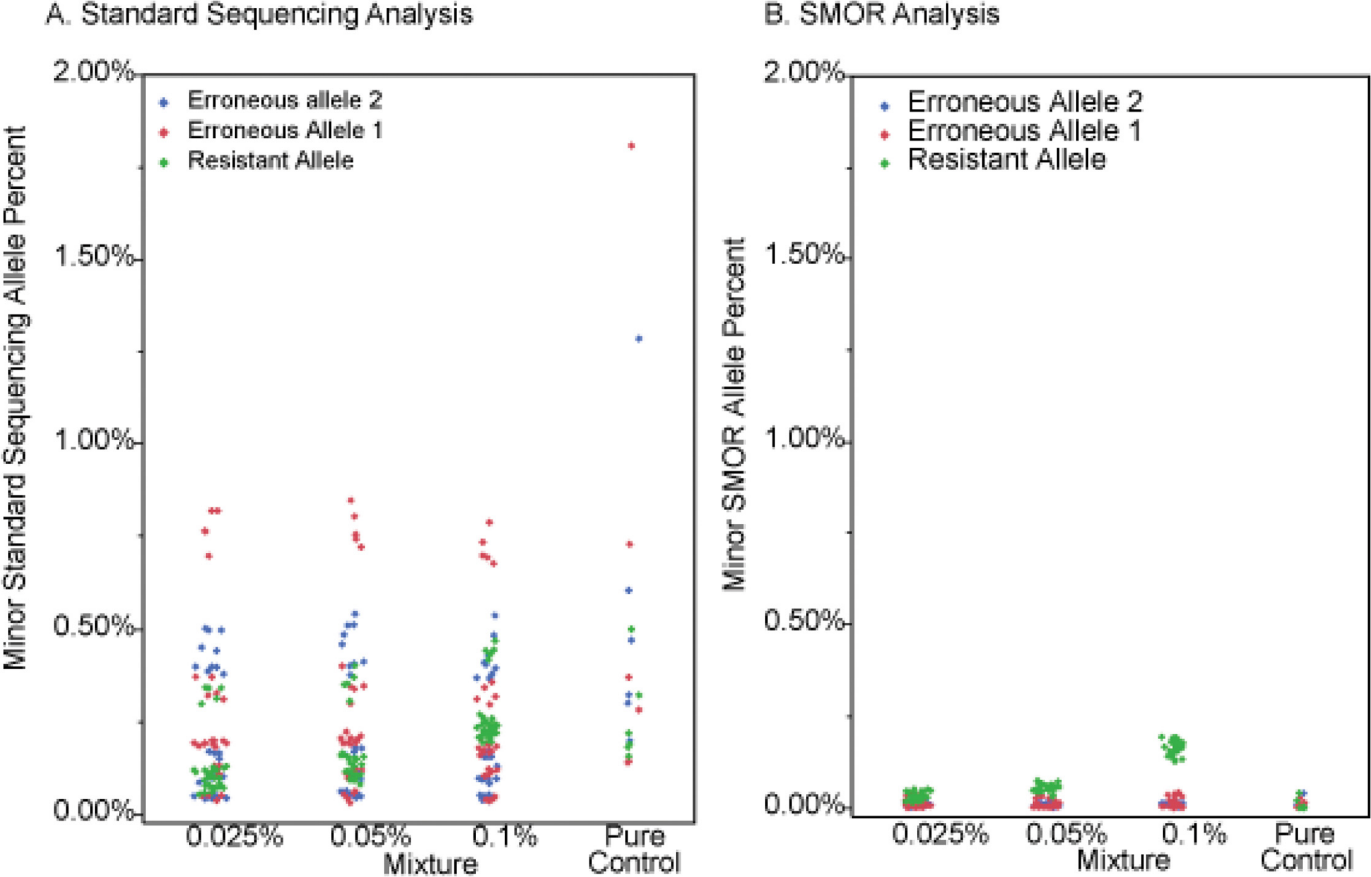
Comparison of standard sequencing and SMOR analysis. The percentage of base calls for the resistant allele compared to the erroneous alleles at six resistant SNP loci in five different genes, five replicates each, is shown. All 36 SNP loci were examined; however most SNP loci are consistent between the resistant and susceptible strains used in the mixtures. The seven different mixtures contain six known allelic differences in resistant conferring loci. Each circle represents the percent of calls for a particular allele for each replicate and the color represents the type of allele. The pure control was the pan susceptible isolate DNA 2–0112. A. Results from standard sequencing analysis, ignoring read pair information. B. Results from SMOR analysis. For the means and standard deviations see S6 Table.

The SMOR analysis approach utilized small amplicons, universal-tail PCR, and complete overlapping reads to detect and quantify drug resistance conferring SNPs in six different target gene regions (*katG*, *inhA* promoter, *and rpoB*, *gyrA*, *rrs*, and *eis* promoter) in mixed subpopulations of resistant to susceptible *M*. *tuberculosis*. Artificial mixtures of an extensively drug resistant (XDR) strain of *M*. *tuberculosis* and a pan-susceptible *M*. *tuberculosis* strain were examined; with the XDR strain contributing from 0.025% to 70% of the mixture. The mixtures were developed with normalized standards, and reproducibility was proven using five replicates. SMOR analysis was compared to standard NGS sequencing analysis and resulted in an increase in sensitivity with the SMOR approach (Fig 2). The pure susceptible culture was used to examine erroneous allele frequencies, at all positions across each amplicon, and serve as a control on each sequencing run. With standard sequencing NGS analysis, the three erroneous allele mean frequencies across the six known differences in SNP loci examined in the *in vitro* mixtures from pure culture ranged from 0.26% (+/-0.13%) to 0.58% (+/-0.64%), meaning detection of subpopulations below these levels was not possible. Conversely, the SMOR erroneous mean frequencies from pure culture ranged from 0.011% (+/-0.016%) to 0.013% (+/-0.019%), more than an order of magnitude below the standard NGS erroneous call rates (S6 Table). In both the standard and SMOR analyses, constant within-target erroneous SNP allele call frequencies were observed, while error levels among targets varied, illustrating a consistent level of intrinsic error for each analysis approach (Table 1).

**Table 1:** Comparison of observed error rate for SMOR analysis versus standard NGS analysis on a pure pan-susceptible *Mycobacterium tuberculosis* isolate across the entire amplicon.

	SMOR Analysis		Standard NGS Analysis	
Gene target	Average Error Rate	Standard Deviation	Average Error Rate	Standard Deviation
*katG*	3.67x10^-04^	2.23x10^-04^	1.04x10^-02^	8.89x10^-03^
*inhA* promoter	3.52x10^-04^	2.39x10^-04^	1.05x10^-02^	1.15x10^-02^
*rpoB*	3.67x10^-04^	2.13x10^-04^	1.05x10^-02^	7.54x10^-03^
*gyrA*	3.66x10^-04^	2.27x10^-04^	9.66x10^-03^	8.70x10^-03^
*rrs*	3.41x10^-04^	2.83x10^-03^	7.48x10^-03^	5.45x10^-03^
*eis* promoter	4.78x10^-04^	4.43x10^-04^	2.31x10^-02^	2.78x10^-02^

**Fig 3:**
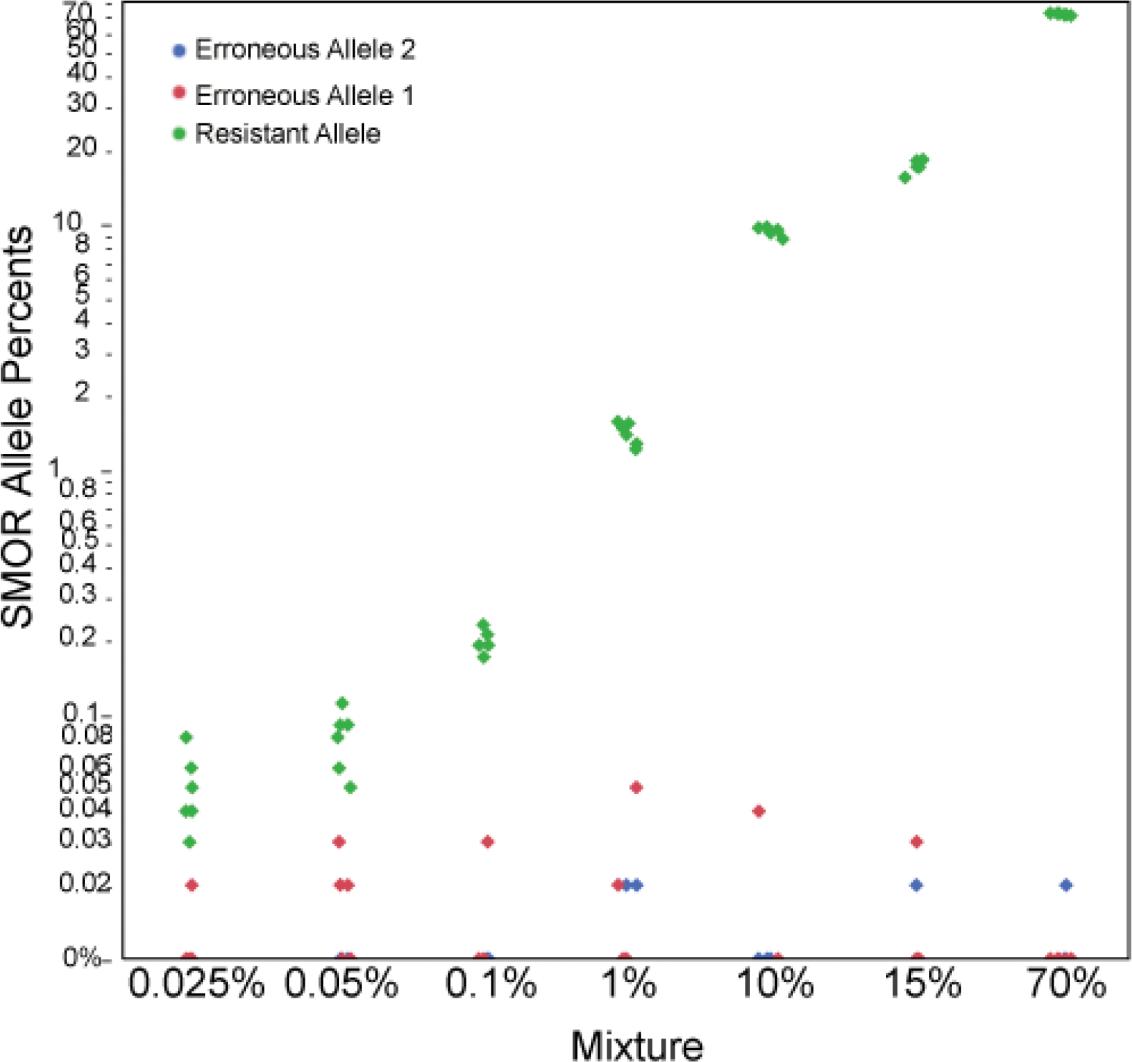
SMOR minor subpopulation examination of a single replicate of 7 mixtures at six known differing resistance SNP loci. Each circle represents the percent SMOR call, where color represents allele state, for a single sample at the six resistant SNP loci. All 36 SNP loci were examined; however the seven different mixtures contain six known allelic differences in resistant conferring SNP loci.

**Fig 4:**
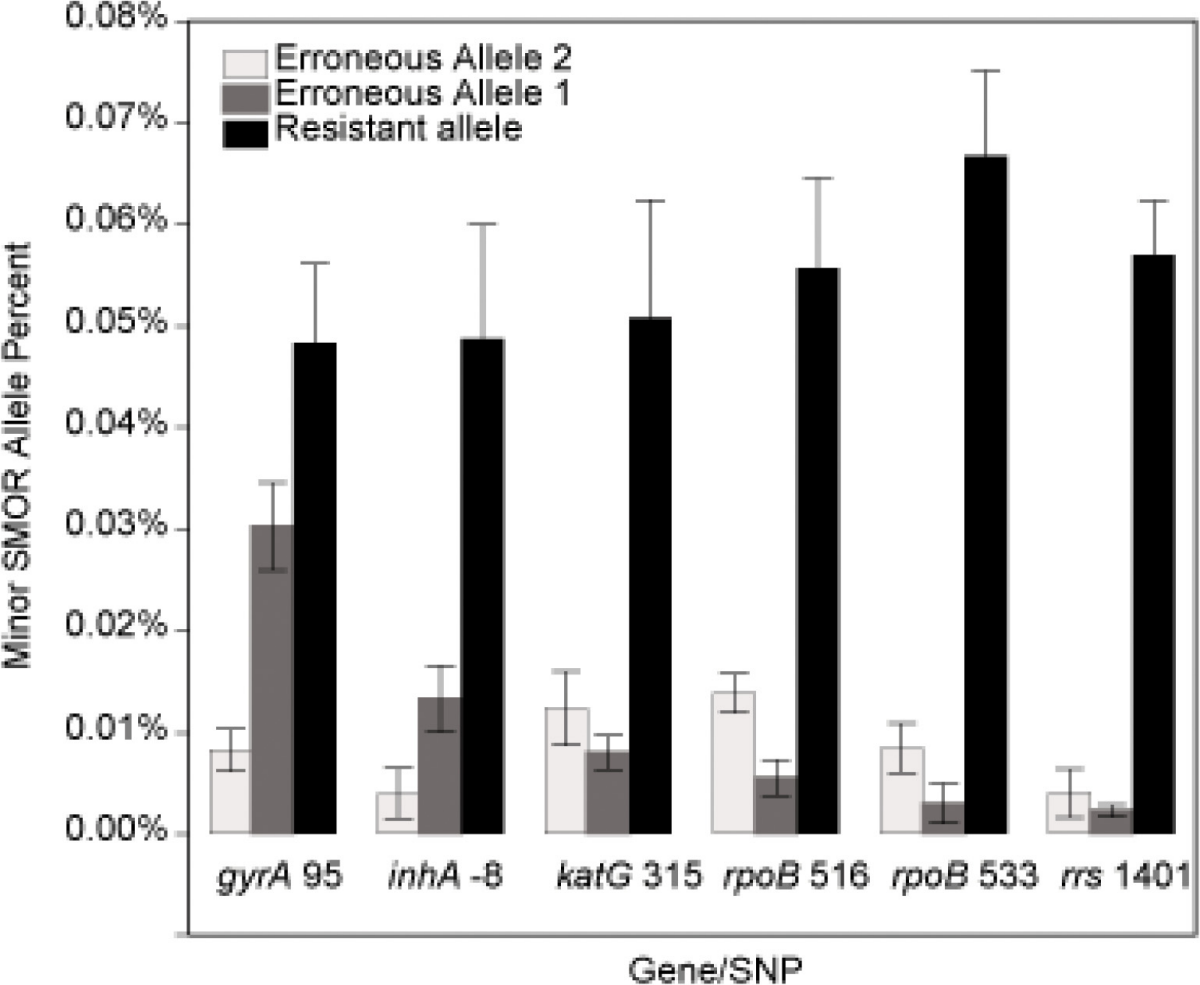
Variation of erroneous alleles. Average frequency (five replicates) of the resistant allele compared to the erroneous alleles at six specific SNP loci from the 0.05:99.95 XDR:Pan-susceptible mixture. Each error bar is constructed using 1 standard deviation from the mean.

In the validation studies, the mean paired read coverage across the six gene targets within a sample was 1.31 x 10^5^ paired reads (SD 8.78 x 10^3^), which would allow for a theoretical limit of detection as low as 0.008% subpopulation based on a threshold of having at least 10 read pairs belonging to the minor subpopulation. The assay resulted in consistent coverage depth across gene targets (column labeled #Hom in S5 Table has the coverage in the number of read pairs), along with consistent estimated resistant allele frequencies within mixtures (Fig 3). Consistency across replicates was also obtained, illustrating the robustness of the assay (Fig 4, and S6 Table).

The *M*. *tuberculosis* specific primers performed with 100% specificity across a diversity panel examining clinically relevant organisms (S4 Table). None of the non-target DNA samples, including dozens of differential diagnostic species, amplified. The addition of excess (~2,000 fold, based on mass) human DNA to the *M*. *tuberculosis* isolate DNA did not have a measurable effect on the PCR efficiency. Additionally none of the three *M*. *tuberculosis*-negative human sputum samples analyzed showed any *M*. *tuberculosis* specific amplification. One *M*. *tuberculosis*-negative human sputum sample had a single non-*M*. *tuberculosis* amplification product and was easily excluded from analysis based upon fragment size and sequence divergence from *M*. *tuberculosis*.

Sample processing was evaluated using a dilution series of the 10:90 XDR to pan-susceptible TB mixture. By increasing the number of cycles in the index extension PCR step, from the standard 6 cycles to 16 cycles, amplification was achieved down to 500 fg (~250 genomic copies of *M*. *tuberculosis*) resulting in libraries of appropriate concentration for sequencing. To examine the PCR effect on variation of resistant alleles across resistant SNP loci known to be present, an increased concentrated sample (50 pg 10% XDR-TB) was run with the extended cycle parameters. This allowed for an examination of the effect of additional 10 PCR cycles on the erroneous base calls for these positions (S1 Fig). By using a high fidelity polymerase, PCR cycles could be added without a significant increase in error (one-way ANOVA, *F*(1,22) = 0.2830, *p* = 0.6001), and no difference in resistant allele frequencies with extended cycles on the 50 pg sample was identified. However, significant variation in the resistant allele frequencies with the 500 fg sample (2-sided F test of equal variance *p* = 0.008) was found, which was attributed to stochastic primer effects due to extremely low input DNA amounts.

### Application: Clinical samples

**Fig 5:**
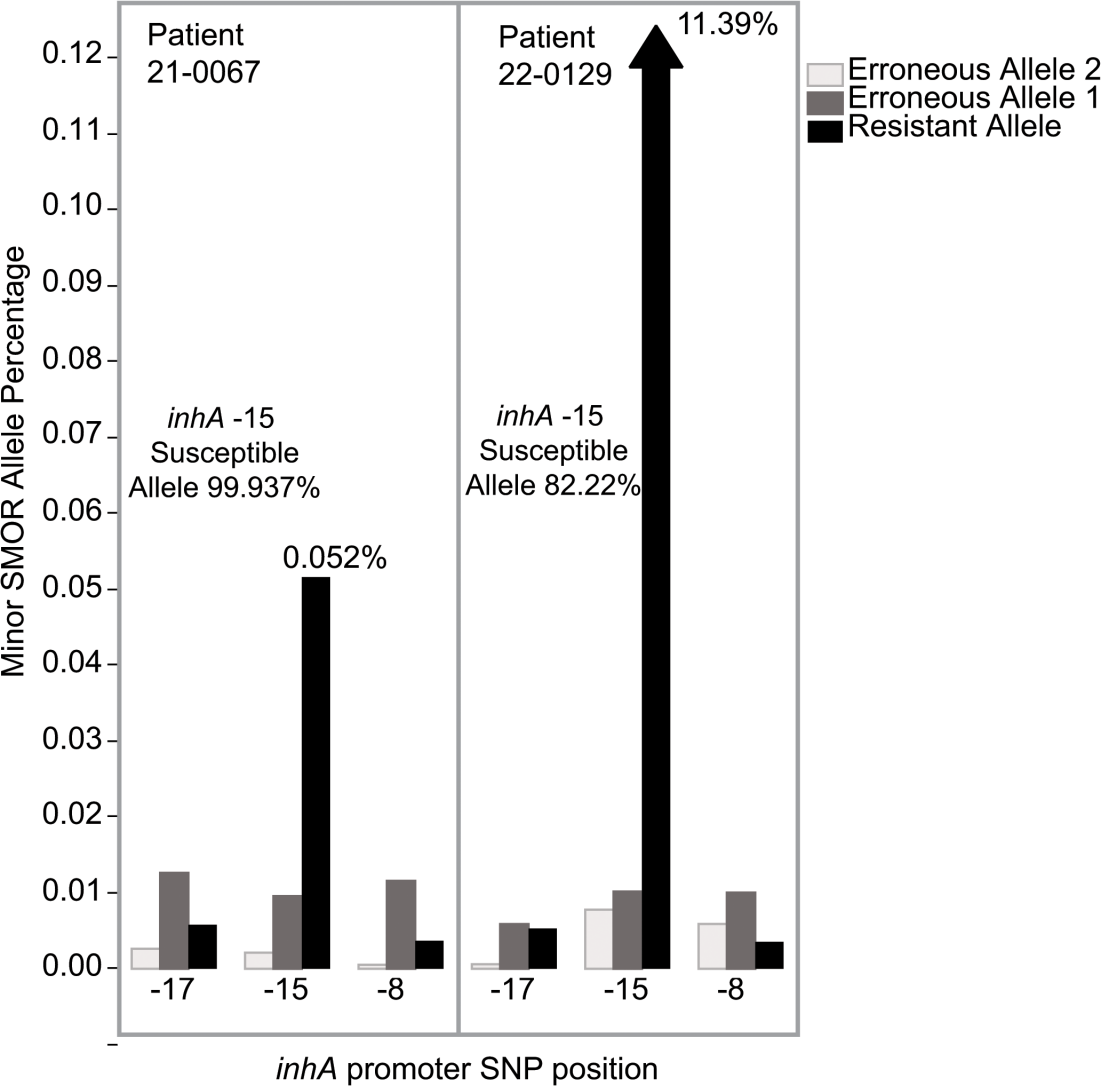
Minor subpopulation detection in two sputum samples from Moldova. Resistant and erroneous allele frequencies from three resistance SNP loci in the *inhA* promoter are shown. Patient 21–0067 with 0.05% resistant allele and patient 22–0129 with 11.39% resistant allele at *inhA* -15, compared to erroneous and resistant alleles below 0.01% at the other two SNP positions.

In order to demonstrate the practical utility of SMOR analysis with clinical samples, DNA from a convenient sample of 12 sputum samples collected from *M*. *tuberculosis* patients in Moldova were subjected to SMOR analysis. Due to the limited amount of DNA available from these samples, an increased number of PCR amplification cycles were necessary to yield sufficient amplicon material for sequencing (see Methods section). One sample failed to amplify, presumably due to insufficient input target DNA. SMOR analysis was used to characterize all 36 resistance loci in the 11 remaining samples. The genotypic resistance profile closely matched the phenotypic resistance profile (S7 Table). Initial Kanamycin discordance for sample 21–0100 was resolved by expanding the SMOR analysis to include position -37 in the *eis* promoter, the G-T mutation, a previously published resistance conferring SNP[45]. Where limited discordance was identified between SMOR and phenotypic DST (sample 21–0017), the SMOR call was determined by the major population being resistant or susceptible (S8 Table). This major population call was confirmed by separate pyrosequencing of target loci (data not shown). Within the analyzed clinical samples, limited minor subpopulations of resistance SNPs were identified. For example, resistance allele subpopulations at 11% and 0.05% of the same *inhA* promoter resistance locus (*inhA* -15) were found in two of the clinical samples, whereas resistance alleles at the other two known SNP loci (*inhA* -8, and -17) within that gene were consistent with erroneous allele calls (~0.01%) (Fig 5). Furthermore, patient 22–0129 had a resistance allele in katG315 at the same allelic frequency (11.7%).

## Discussion

NGS has vastly improved nucleic acid sequencing with respect to amount, speed, and cost of data obtained. There are numerous potential clinical applications of NGS; one of the most promising currently is amplicon sequencing of clinically relevant genetic targets, such as human genes known to be involved in disease or microbial pathogen targets. Examination of population diversity with deep sequencing has been an emerging focus in both viral[27, 33, 46, 47] and antibody[32, 48] research. Targeted NGS amplicon sequencing can provide high levels of sample and target multiplexing, leading to simultaneous detection and characterization of dozens to hundreds of genetic targets in <24 hours, instead of days to weeks. Amplicon sequencing can be applied to low levels of degraded DNA, which is often the case with clinical samples. Lastly, amplicon sequencing, due to the ability to obtain extremely deep levels of coverage across the targeted amplicons, has the potential for detecting ultra-rare genetic variant subpopulations[32, 47, 48]. In a clinical setting, this approach could lead to early detection and monitoring of antibiotic resistant subpopulations, which, if left undetected, could lead to selection of the resistant population and ultimately, treatment failure.

The amount of sequencing coverage needed for detection is set by the frequency of the subpopulation to be examined. For example, detection of a 1% subpopulation would require a minimum of 1,000x of paired reads (2,000x total coverage) in order to observe10 pairs of reads representing the minor (e.g., resistant) subpopulation. The coverage requirement, therefore, is inversely proportional to the subpopulation percentage detection requirement. Utilizing an amplification-based approach enables ultra-rare subpopulation detection in the presence of minimal bacterial loads, which are approximated by very low *M*. *tuberculosis* DNA copies or genome equivalents. This is imperative for clinical sample analysis. The *M*. *tuberculosis* bacterial load can be very low within the patient sputa sample (<1000 cfu/ml), often requiring culture of the bacteria to run clinical tests, including phenotypic susceptibility analyses. The ability to characterize and quantify drug resistant subpopulations directly from clinical samples, with minimal DNA input, is a dramatic step toward the real-time management of drug resistant TB; however, there are caveats. With very low input DNA stochastic PCR effects will increase the variance among different gene estimations of resistant allele frequencies. Based on the analysis of the 10% mixture 500fg (~250 genome copies) sample (S1 Fig), while detection of a subpopulation is possible, estimates of the variant allele frequency will vary greatly across SNP targets. To examine the probability that at least one genome from the resistant subpopulation is added to the Gene Specific PCR, given a total number of genomes added to the reaction, the Poisson distribution was used. To have 95% confidence that one or more genome of the resistant subpopulation is added of a 0.05% mixture, 10,000 genomes must be present in the original PCR. An initial estimation of *M*. *tuberculosis* genome copy number (e.g., smear counts or qPCR) in a given sample will guide optimal sample amplification parameters as well as prevent false negative conclusions about the absence of low-level resistant subpopulations due to low template input.

The specificity of the assay targets is crucial in a clinical setting, especially in the absence of an isolation culturing step, as DNA from other organisms as well as human will be present and can compound the difficulty of examining the *M*. *tuberculosis* signal. The assay primers used here were found to be 100% specific across a diversity panel of clinically relevant DNA (S4 Table) and no discernable reduction in PCR efficiency was seen in the presence of excess human DNA.

The novel universal-tail indexing and ligation free amplicon library preparation methodology presented here has several benefits, for example, the ability to increase the number of samples in a sequencing pool at a low cost (i.e., without universal-tail indexing one would need a different indexed primer for every target and every sample, such as in previously described 16S and antibody amplicon sequencing methodologies[32, 49]). Furthermore this method allows for the easy addition of targets to an existing assays system or the rapid development of new target organism systems. The universal tail primers can theoretically be used on any organism and indeed has been tested on a number of diverse bacterial organisms (data not shown), thus cutting down the startup cost for new pathogens. Once the extension indices are purchased they can be used across organisms, samples, and targets in numerous combinations. The simplification of the library preparation to just two PCR steps, reduces the error associated with ligation and other standard library preparation processes[32]. The SMOR system has the potential for rapidly developing a number of clinically relevant applications for table-top sequencing of other rare variant detection needs, from forensic analysis to cancer detection.

The rapid turnaround with the universal tail method is a vast improvement over traditional methods, beyond just time-to-answer; not only is an extensive genetic resistance profile produced, but also quantitative analysis of resistant populations. The determination of the ratio of resistant to susceptible alleles will enable intensive examination of both the emergence and importance of low-level genetic resistance throughout treatment. The theory that rapid emergence of resistance is facilitated by highly diverse large populations within host[50] can be examined with this methodology directly on patient samples. Current WGS methods cannot overcome their respective inherent error rates to establish such ratios at minor populations levels below 1% and is especially difficult directly on clinical samples where majority of the sequence reads will not belong to the pathogen in question. Additionally, no method is capable of identifying the time point at which drug resistant subpopulations in clinical samples from tuberculosis patients become clinically relevant across various loci. The use of SMOR allows for the *in situ* examination of dynamic changes of mixed subpopulations over time of *M*. *tuberculosis* infections.

Standard NGS can identify moderately low-level presence of resistance conferring SNP allele in a mixture of resistant and susceptible populations. However, detecting ultra-rare subpopulations is limited by several factors, most importantly the intrinsic sequencing error rate. SMOR analysis was designed to address the complexity of detecting SNPs at or below the frequency of standard sequencing error. This is achieved by exploiting the longer paired sequence reads of current sequencing technology to independently interrogate both strands of a target locus from the same DNA molecule (S2 Fig), providing independent confirmation of specific nucleotides at any given position within the sequenced amplicon. With standard sequencing NGS analysis, the detection of a resistant subpopulation < 2% would be difficult due to inherent error rates (Fig 2). However, while erroneous call rates will vary among genetic regions due to characteristics of specific loci and surrounding nucleotide sequence, with SMOR analysis detection of resistant subpopulations ≥0.1% is now attainable (Figs 2 and 4, and S3 Fig). Also, while the clinical relevance of ultra-low (<1%) subpopulations in *Mtb* has yet to be ascertained, even lower detections limits may be possible with SMOR analysis. One significant hurdle is sample index cross contamination during sequencing, resulting in low level mixing of sample reads, most often due to mixed fragment clusters or optical cluster address misidentification on the flow cell. These issues can be addressed with stringent index read quality filtering and sample dual indexing[51], and are currently be evaluated with the *Mtb* assays system presented here.

## Conclusions

The amplicon sequencing/SMOR method presented here promises significant advantages over existing methodologies for detection of clinically relevant genetic targets, especially when present at low levels within the infecting population. It allows for rapid and cost effective examination of known resistance conferring SNPs in multiple gene targets simultaneously, at detection levels at or lower than current methods. Additionally, previously undetectable ultra-rare resistant subpopulations (<1%) can now be characterized and evaluated for clinical significance. For the current analysis, clinical samples could be examined at all 6 gene targets to at least 50,000x coverage in 72 hours at a cost of ~$30/sample in reagents, but this could feasibly be lowered to ~24 hours and $10/sample in reagents depending on throughput and assay design. The amplicon/SMOR assay can be expanded as new resistance markers are identified without much additional work, overall cost, or additional time. The application of amplicon/SMOR methodology on actual TB patient sputum samples highlights this clinical practicality. As bench-top sequencers, with both local and cloud-based tools, are becoming more readily available, this method has great promise for rapid comprehensive detection and quantification of drug resistance in clinical samples.

## Supporting Information

S1 Fig Effects of DNA input concentration. Frequency of the susceptible, resistant and erroneous alleles from a single sample, averaged across 5 different genes. All 36 loci were examined; however the mixture contains six known allelic differences in resistant conferring loci. Each error bar is constructed using 1 standard deviation from the mean.
(DOCX)

S2 Fig SMOR allele calls versus single read errors. Alternating shading illustrates paired reads aligned to reference genome. The black arrow indicates a pair of reads where both reads from the one DNA molecule have the same alternate base, representing a true minor component. The white arrow indicates where one read in a pair has an alternate base, representing a sequencing error.
(DOCX)

S3 Fig Minor component analysis across all 36 SNP loci for 0.1% mixture. SNP loci are designated by either the position in the promoter or the codon for each gene target, and correspond to genomic positions in S2 Table.
(DOCX)

S1 Table *M*. *tuberculosis* specific primer with universal tail sequences. All oligos are with standard de-salting. The universal tail sequences are highlighted in red with the forward primer sequence differing from the reverse primer sequence.
(DOCX)

S2 Table Antibiotic resistance conferring SNP genomic positions. (DOCX)

S3 Table Illumina index extension primers and sequencing primers. All oligos listed have been validated and were synthesized with standard de-salting.
(DOCX)

S4 Table Diversity DNA panel the gene specific primers were tested against. (DOCX)

S5 Table SMOR script output. (XLSX)

S6 Table Comparison of standard NGS and SMOR analyses: means and standard deviations. (DOCX)

S7 Table Comparison of MIDGT DST and SMOR analysis. (DOCX)

S8 Table SMOR results of major population from clinical samples. (DOCX)
