# Supplementary material for: Detection of Low-Level Mixed-Population Drug Resistance in Mycobacterium tuberculosis Using High Fidelity Amplicon Sequencing
Source: PLoS One. 2015 May 13;10(5):e0126626. doi: 10.1371/journal.pone.0126626 (PMC4430321; doi:10.1371/journal.pone.0126626)
Supplement: S1 Fig — Frequency of the susceptible, resistant and erroneous alleles from a single sample, averaged across 5 different genes. All 36 loci were examined; however the mixture contains six known allelic differences in resistant conferring loci. Each error bar is constructed using 1 standard deviation from the mean. (DOCX) [file pone.0126626.s001.docx]

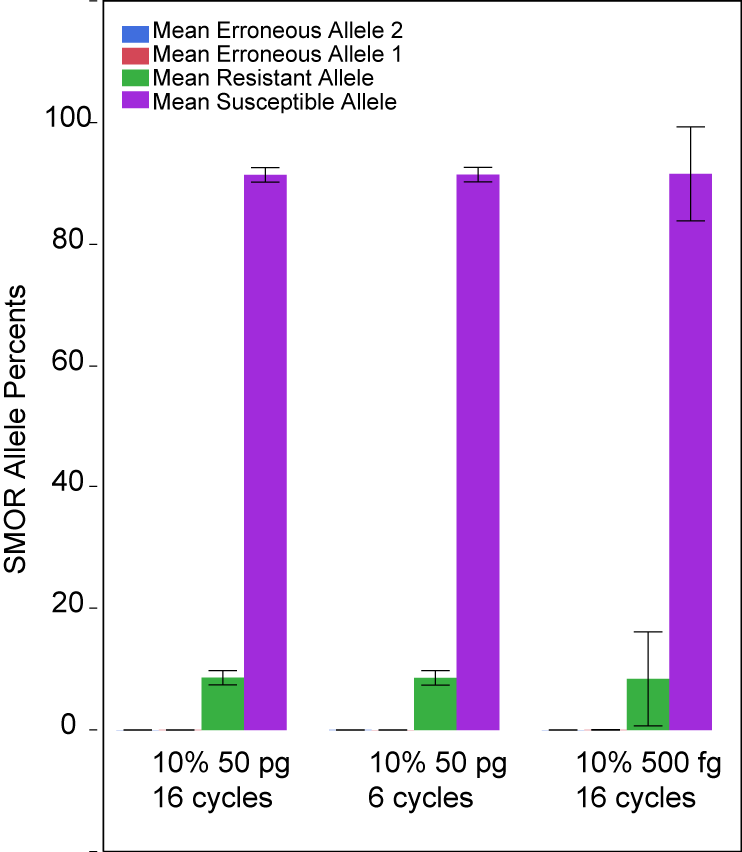


**Figure S1. Effects of DNA input concentration.** Frequency of the susceptible, resistant and erroneous alleles from a single sample, averaged across 5 different genes. All 36 loci were examined; however the mixture contains six known allelic differences in resistant conferring loci. Each error bar is constructed using 1 standard deviation from the mean.
