# Supplementary material for: Detection of Low-Level Mixed-Population Drug Resistance in Mycobacterium tuberculosis Using High Fidelity Amplicon Sequencing
Source: PLoS One. 2015 May 13;10(5):e0126626. doi: 10.1371/journal.pone.0126626 (PMC4430321; doi:10.1371/journal.pone.0126626)
Supplement: S2 Fig — Alternating shading illustrates paired reads aligned to reference genome. The black arrow indicates a pair of reads where both reads from the one DNA molecule have the same alternate base, representing a true minor component. The white arrow indicates where one read in a pair has an alternate base, representing a sequencing error. (DOCX) [file pone.0126626.s002.docx]

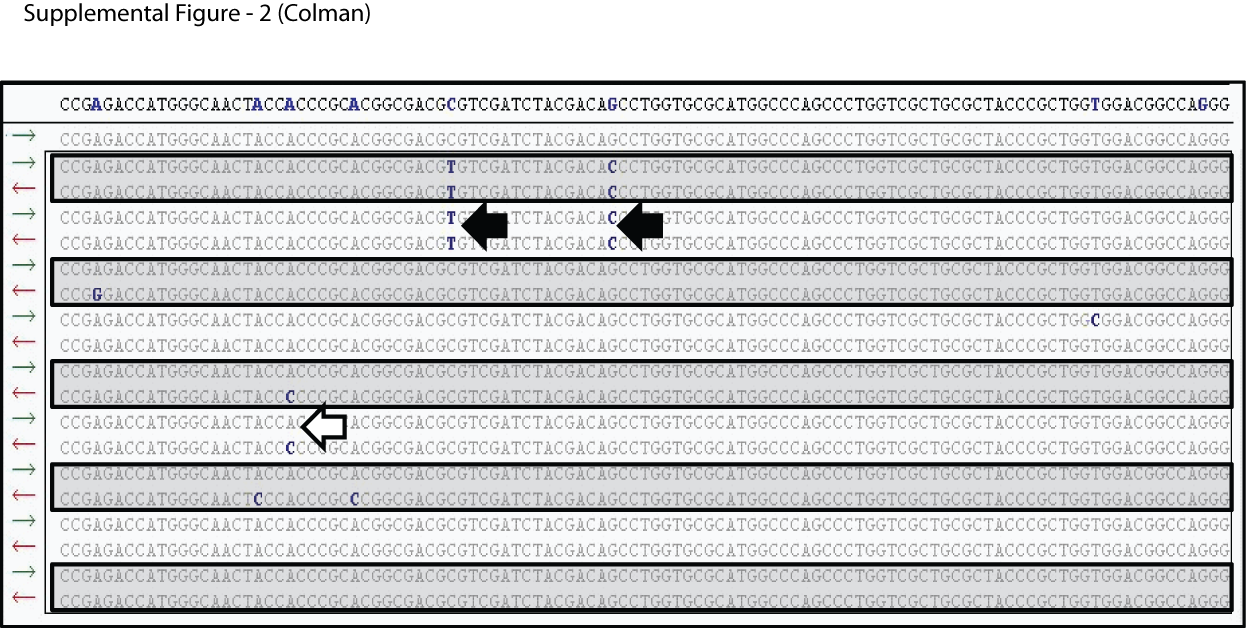


**Figure S2. SMOR allele calls versus single read errors.** Alternating shading illustrates paired reads aligned to reference genome. The black arrow indicates a pair of reads where both reads from the one DNA molecule have the same alternate base, representing a minor component. The white arrow indicates where one read in a pair has an alternate base, representing a sequencing error.
