# Supplementary material for: Detection of Low-Level Mixed-Population Drug Resistance in Mycobacterium tuberculosis Using High Fidelity Amplicon Sequencing
Source: PLoS One. 2015 May 13;10(5):e0126626. doi: 10.1371/journal.pone.0126626 (PMC4430321; doi:10.1371/journal.pone.0126626)
Supplement: S3 Fig — SNP loci are designated by either the position in the promoter or the codon for each gene target, and correspond to genomic positions in S2 Table. (DOCX) [file pone.0126626.s003.docx]

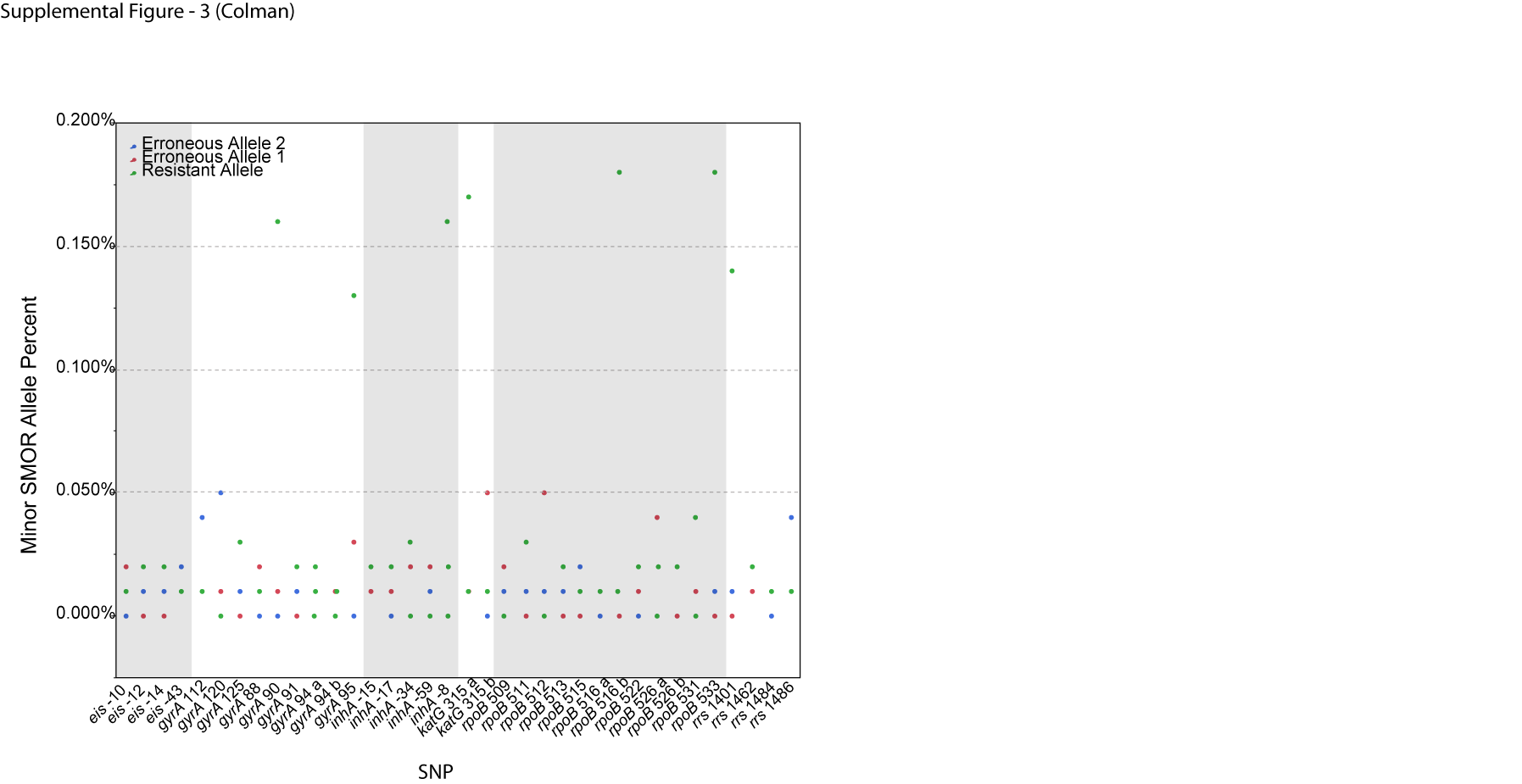


**Figure S3. Minor component analysis across all 36 SNP loci for 0.1% mixture.** SNP loci are designated by either the position in the promoter or the codon for each gene target, and correspond to genomic positions in Supplemental Table S2.
