# Supplementary material for: Detection of Low-Level Mixed-Population Drug Resistance in Mycobacterium tuberculosis Using High Fidelity Amplicon Sequencing
Source: PLoS One. 2015 May 13;10(5):e0126626. doi: 10.1371/journal.pone.0126626 (PMC4430321; doi:10.1371/journal.pone.0126626)
Supplement: S1 Table — All oligos are with standard de-salting. The universal tail sequences are highlighted in red with the forward primer sequence differing from the reverse primer sequence. (DOCX) [file pone.0126626.s004.docx]

**Table S1. *M. tuberculosis* specific primer with universal tail sequences.** All oligos are with standard de-salting. The universal tail sequences are highlighted in red and bold with the forward primer sequence differing from the reverse primer sequence.

| **Forward Primer** | **sequence** |
| --- | --- |
| gyrAv2fUT1 | **ACCCAACTGAATGGAGC**GGGTGCTCTATGCAATGTTCGAT |
| eisv2fUT1 | **ACCCAACTGAATGGAGC**CGTCAACCGCAGATCCATGTAC |
| rpoBv2fUT1 | **ACCCAACTGAATGGAGC**CGATCACACCGCAGACGTT |
| katGv2fUT1 | **ACCCAACTGAATGGAGC**CCATGAACGACGTCGAAACAG |
| inhAv2fUT1 | **ACCCAACTGAATGGAGC**CCTCGCTGCCCAGAAAGG |
| rrsv2fUT1 | **ACCCAACTGAATGGAGC**CTAGTAATCGCAGATCAGCAACG |
| **Reverse Primer** | **sequence** |
| gyrAv2rUT2 | **ACGCACTTGACTTGTCTTC**GGGCTTCGGTGTACCTCATC |
| eisv2rUT2 | **ACGCACTTGACTTGTCTTC**CGTCGCTGATTCTCGCAGTG |
| rpoBv2rUT2 | **ACGCACTTGACTTGTCTTC**GTTTCGATCGGGCACATCC |
| katGv2rUT2 | **ACGCACTTGACTTGTCTTC**GCTCTTCGTCAGCTCCCACTC |
| inhAv2rUT2 | **ACGCACTTGACTTGTCTTC**GTCACATTCGACGCCAAACAG |
| rrsv2rUT2 | **ACGCACTTGACTTGTCTTC**GCCTACGCCCCACCAGTT |
|  |  |
