# Supplementary material for: Detection of Low-Level Mixed-Population Drug Resistance in Mycobacterium tuberculosis Using High Fidelity Amplicon Sequencing
Source: PLoS One. 2015 May 13;10(5):e0126626. doi: 10.1371/journal.pone.0126626 (PMC4430321; doi:10.1371/journal.pone.0126626)
Supplement: S2 Table — (DOCX) [file pone.0126626.s005.docx]

**Table S2. Antibiotic resistance conferring SNP genomic positions.**

| **gene** | **codon/**  **nucleotide** | **mutation** | **codon position in H37Rv** | **SNP location in H37Rv** | **Wild type** | **Mutant** |
| --- | --- | --- | --- | --- | --- | --- |
| *eis* | -10 | G-A | --- | 2715344 | C* | T* |
| *eis* | -12 | C-T | --- | 2715346 | G* | A* |
| *eis* | -14 | C-T | --- | 2715375 | G* | A* |
| *eis* | -43 | A-T | --- | 2715342 | T* | A* |
| *gyrA* | 88 | GGC-TGC | 7563-7565 | 7563 | G | T |
| *gyrA* | 90 | GCG-GTG | 7569-7571 | 7570 | C | T |
| *gyrA* | 91 | TCG-CCG | 7572-7574 | 7572 | T | C |
| *gyrA* | 94 a | GAC-(A/C/T)AC | 7581-7583 | 7581 | G | A/C/T |
| *gyrA* | 94 b | GAC-G(C/G)C | 7581-7583 | 7582 | A | C/G |
| *gyrA* | 95** | AGC-ACC | 7584-7586 | 7585 | G | C |
| *gyrA* | 112** | GGC-GGG | 7635-7637 | 7637 | C | G |
| *gyrA* | 120** | GGC-GGG | 7659-7661 | 7661 | C | G |
| *gyrA* | 125** | GCG-GCA | 7674-7676 | 7676 | G | A |
| *inhA* | -59 | G-C | --- | 1673381 | G | C |
| *inhA* | -34 | C-G/T | --- | 1673406 | C | G/T |
| *inhA* | -17 | G-T | --- | 1673423 | G | T |
| *inhA* | -15 | C-T | --- | 1673425 | C | T |
| *inhA* | -8 | T-A/C/G | --- | 1673432 | T | A/C/G |
| *katG* | 315 b | AGC-ACA | 2155167-2155169 | 2155167 | G* | T* |
| *katG* | 315 a | AGC-A(A/C)C | 2155167-2155169 | 2155168 | C* | G/T* |
| *rpoB* | 509** | AGC-CGC | 761088-761090 | 761088 | A | C |
| *rpoB* | 511** | CTG-CCG | 761094-761096 | 761095 | T | C |
| *rpoB* | 512** | AGC-ACC | 761097-761099 | 761098 | G | C |
| *rpoB* | 513 | CAA-AAA | 761100-761102 | 761100 | C | A |
| *rpoB* | 515** | ATG-ATA | 761106-761108 | 761108 | G | A |
| *rpoB* | 516 a | GAC-TAC | 761109-761111 | 761109 | G | T |
| *rpoB* | 516 b | GAC-G(G/T)C | 761109-761111 | 761110 | A | G/T |
| *rpoB* | 522 | TCG-TTG | 761127-761129 | 761128 | C | T |
| *rpoB* | 526 a | CAC-(G/T)AC | 761139-761141 | 761139 | C | G/T |
| *rpoB* | 526 b | CAC-TAC | 761139-761141 | 761140 | A | G |
| *rpoB* | 531 | TCG-T(T/G)G | 761154-761156 | 76115 | C | T/G |
| *rpoB* | 533** | CTG-CCG | 761160-761162 | 761161 | T | C |
| *rrs* | 1401 | A-G | --- | 1473246 | A | G |
| *rrs* | 1462** | A-T | --- | 1473307 | A | T |
| *rrs* | 1484 | G-T | --- | 1473329 | G | T |
| *rrs* | 1486** | A-T | --- | 1473331 | A | T |

*SNP is described on the negative strand

**SNP not confirmed to confer resistance, possible positions of interest or phylogenetic informative
