# Supplementary material for: Detection of Low-Level Mixed-Population Drug Resistance in Mycobacterium tuberculosis Using High Fidelity Amplicon Sequencing
Source: PLoS One. 2015 May 13;10(5):e0126626. doi: 10.1371/journal.pone.0126626 (PMC4430321; doi:10.1371/journal.pone.0126626)
Supplement: S3 Table — All oligos listed have been validated and were synthesized with standard de-salting. (DOCX) [file pone.0126626.s006.docx]

**Table S3. Illumina index extension primers and sequencing primers.** All oligos listed have been validated and were synthesized with standard de-salting

| **Indexing extension primers** | **sequence** |
| --- | --- |
| Common Illumina-UT1 | AATGATACGGCGACCACCGAGATCTACACTATGGTAATTGTACCCAACTGAATGGAGC |
| Indexed Illumina-UT2-1 | CAAGCAGAAGACGGCATACGAGATACAAGCTAAGTCAGTCAGCCACGCACTTGACTTGTCTTC |
| Indexed Illumina-UT2-2 | CAAGCAGAAGACGGCATACGAGATAAACATCGAGTCAGTCAGCCACGCACTTGACTTGTCTTC |
| Indexed Illumina-UT2-3 | CAAGCAGAAGACGGCATACGAGATACATTGGCAGTCAGTCAGCCACGCACTTGACTTGTCTTC |
| Indexed Illumina-UT2-4 | CAAGCAGAAGACGGCATACGAGATACCACTGTAGTCAGTCAGCCACGCACTTGACTTGTCTTC |
| Indexed Illumina-UT2-5 | CAAGCAGAAGACGGCATACGAGATAACGTGATAGTCAGTCAGCCACGCACTTGACTTGTCTTC |
| Indexed Illumina-UT2-6 | CAAGCAGAAGACGGCATACGAGATCGCTGATCAGTCAGTCAGCCACGCACTTGACTTGTCTTC |
| Indexed Illumina-UT2-7 | CAAGCAGAAGACGGCATACGAGATCAGATCTGAGTCAGTCAGCCACGCACTTGACTTGTCTTC |
| Indexed Illumina-UT2-8 | CAAGCAGAAGACGGCATACGAGATATGCCTAAAGTCAGTCAGCCACGCACTTGACTTGTCTTC |
| Indexed Illumina-UT2-10 | CAAGCAGAAGACGGCATACGAGATAGTACAAGAGTCAGTCAGCCACGCACTTGACTTGTCTTC |
| Indexed Illumina-UT2-11 | CAAGCAGAAGACGGCATACGAGATCATCAAGTAGTCAGTCAGCCACGCACTTGACTTGTCTTC |
| Indexed Illumina-UT2-12 | CAAGCAGAAGACGGCATACGAGATAGTGGTCAAGTCAGTCAGCCACGCACTTGACTTGTCTTC |
| Indexed Illumina-UT2-13 | CAAGCAGAAGACGGCATACGAGATAACAACCAAGTCAGTCAGCCACGCACTTGACTTGTCTTC |
| Indexed Illumina-UT2-37 | CAAGCAGAAGACGGCATACGAGATCCGAAGTAAGTCAGTCAGCCACGCACTTGACTTGTCTTC |
| Indexed Illumina-UT2-38 | CAAGCAGAAGACGGCATACGAGATCCGTGAGAAGTCAGTCAGCCACGCACTTGACTTGTCTTC |
| Indexed Illumina-UT2-39 | CAAGCAGAAGACGGCATACGAGATCCTCCTGAAGTCAGTCAGCCACGCACTTGACTTGTCTTC |
| Indexed Illumina-UT2-40 | CAAGCAGAAGACGGCATACGAGATCGAACTTAAGTCAGTCAGCCACGCACTTGACTTGTCTTC |
| Indexed Illumina-UT2-41 | CAAGCAGAAGACGGCATACGAGATCGACTGGAAGTCAGTCAGCCACGCACTTGACTTGTCTTC |
| Indexed Illumina-UT2-42 | CAAGCAGAAGACGGCATACGAGATCGCATACAAGTCAGTCAGCCACGCACTTGACTTGTCTTC |
| Indexed Illumina-UT2-43 | CAAGCAGAAGACGGCATACGAGATCTCAATGAAGTCAGTCAGCCACGCACTTGACTTGTCTTC |
| Indexed Illumina-UT2-44 | CAAGCAGAAGACGGCATACGAGATCTGAGCCAAGTCAGTCAGCCACGCACTTGACTTGTCTTC |
| Indexed Illumina-UT2-45 | CAAGCAGAAGACGGCATACGAGATCTGGCATAAGTCAGTCAGCCACGCACTTGACTTGTCTTC |
| Indexed Illumina-UT2-46 | CAAGCAGAAGACGGCATACGAGATGAATCTGAAGTCAGTCAGCCACGCACTTGACTTGTCTTC |
| Indexed Illumina-UT2-47 | CAAGCAGAAGACGGCATACGAGATGACTAGTAAGTCAGTCAGCCACGCACTTGACTTGTCTTC |
| Indexed Illumina-UT2-48 | CAAGCAGAAGACGGCATACGAGATGAGCTGAAAGTCAGTCAGCCACGCACTTGACTTGTCTTC |
| Indexed Illumina-UT2-50 | CAAGCAGAAGACGGCATACGAGATGCCACATAAGTCAGTCAGCCACGCACTTGACTTGTCTTC |
| Indexed Illumina-UT2-51 | CAAGCAGAAGACGGCATACGAGATGCGAGTAAAGTCAGTCAGCCACGCACTTGACTTGTCTTC |
| Indexed Illumina-UT2-52 | CAAGCAGAAGACGGCATACGAGATGCTAACGAAGTCAGTCAGCCACGCACTTGACTTGTCTTC |
| Indexed Illumina-UT2-53 | CAAGCAGAAGACGGCATACGAGATGCTCGGTAAGTCAGTCAGCCACGCACTTGACTTGTCTTC |
| Indexed Illumina-UT2-54 | CAAGCAGAAGACGGCATACGAGATGGAGAACAAGTCAGTCAGCCACGCACTTGACTTGTCTTC |
| Indexed Illumina-UT2-55 | CAAGCAGAAGACGGCATACGAGATGGTGCGAAAGTCAGTCAGCCACGCACTTGACTTGTCTTC |
| Indexed Illumina-UT2-56 | CAAGCAGAAGACGGCATACGAGATGTACGCAAAGTCAGTCAGCCACGCACTTGACTTGTCTTC |
| Indexed Illumina-UT2-57 | CAAGCAGAAGACGGCATACGAGATGTCGTAGAAGTCAGTCAGCCACGCACTTGACTTGTCTTC |
| Indexed Illumina-UT2-58 | CAAGCAGAAGACGGCATACGAGATGTCTGTCAAGTCAGTCAGCCACGCACTTGACTTGTCTTC |
| Illumina-UT2-97 (11 rev comp) | CAAGCAGAAGACGGCATACGAGATACTTGATGAGTCAGTCAGCCACGCACTTGACTTGTCTTC |
| Illumina-UT2-98 (58 rev comp) | CAAGCAGAAGACGGCATACGAGATTGACAGACAGTCAGTCAGCCACGCACTTGACTTGTCTTC |
| **Sequencing primers** | **sequence** |
| UT1-R1 seq primer | CCGAGATCTACACTATGGTAATTGTACCCAACTGAATGGAGC |
| UT2-RIndex seq primer | GAAGACAAGTCAAGTGCGTGGCTGACTGACT |
| UT2-R2 seq primer | AGTCAGTCAGCCACGCACTTGACTTGTCTTC |
