# Supplementary material for: Detection of Low-Level Mixed-Population Drug Resistance in Mycobacterium tuberculosis Using High Fidelity Amplicon Sequencing
Source: PLoS One. 2015 May 13;10(5):e0126626. doi: 10.1371/journal.pone.0126626 (PMC4430321; doi:10.1371/journal.pone.0126626)
Supplement: S4 Table — (DOCX) [file pone.0126626.s007.docx]

**Table S4. Diversity DNA panel the gene specific primers were tested against.**

| **Organism** | **Number of isolates** |  | **Organism** | **Number of isolates** |
| --- | --- | --- | --- | --- |
| *Abiotrophia granulicattella grp* | 1 |  | Human gDNA | 2 |
| *Acinetobacter baumannii* | 2 |  | *Legionella pneumophila* | 1 |
| *Acromobacter xylosoxidans* | 1 |  | *Listeria monocytogenes* | 1 |
| *Bacillus spp* | 2 |  | *Micrococcus spp* | 1 |
| *Bacteroides fragilis* | 1 |  | *MRSA* | 2 |
| *Bacteroides uniformis* | 1 |  | *MSSA* | 2 |
| *Bordetella bronchiseptica* | 1 |  | *Mycobacterium abcessus* | 1 |
| *Burkholderia cepacia* | 1 |  | *Mycobacterium avium paratuberculosis* | 2 |
| *Burkholderia pseudomallei* | 3 |  | *Mycobacterium smegmatis* | 1 |
| *Candida albicans* | 1 |  | *Neisseria gonorrhoeae* | 3 |
| *Candida glabrata* | 2 |  | *Neisseria meningitidis* | 3 |
| *Candida parapsilosis* | 3 |  | *Propionibacterium spp* | 1 |
| *Candida tropicalis* | 1 |  | *Providencia stuartii* | 1 |
| *Chryseobacterium indologenes* | 1 |  | *Staphylococcus epidermidis* | 5 |
| *Coccidioides immitis* | 1 |  | *Staphylococcus saprophyticus* | 1 |
| *Coccidioides posadasii* | 2 |  | Coagulase Negative *Staphylococcus* | 6 |
| *Corynebacterium spp* | 1 |  | *Streptococcus agalactiae* | 1 |
| *Coxiella burnetii* | 2 |  | *Streptococcus mitis* | 1 |
| *Enterobacter cloacae* | 9 |  | *Streptococcus pneumoniae* | 4 |
| *Enterococcus faecium* | 2 |  | *Streptococcus pyogenes* | 3 |
| *Enterobacter spp* | 2 |  | *Streptococcus salivarius* | 1 |
| *Enterococcus VRE* | 2 |  | *Streptococcus viridans* grp | 3 |
| *Francisella tulerensis* | 2 |  | Group F *Streptococcus* | 1 |
| *Haemophilus parainfluenzae* | 2 |  | total isolates | 93 |
|  |  |  |  |  |
|  |  |  | *total species* | 47 |
