# Supplementary material for: Detection of Low-Level Mixed-Population Drug Resistance in Mycobacterium tuberculosis Using High Fidelity Amplicon Sequencing
Source: PLoS One. 2015 May 13;10(5):e0126626. doi: 10.1371/journal.pone.0126626 (PMC4430321; doi:10.1371/journal.pone.0126626)
Supplement: S6 Table — (DOCX) [file pone.0126626.s009.docx]

**Table S6. Comparison of standard NGS and SMOR analyses: means and standard deviations.**

| **Standard NGS Analysis** | | |  | |  |  | |  |  | **SMOR Analysis** | | |  |  |  |  |
| --- | --- | --- | --- | --- | --- | --- | --- | --- | --- | --- | --- | --- | --- | --- | --- | --- |
|  | resistant allele | | Erroneous  Allele 1 | | | Erroneous  Allele 2 | | |  |  | resistant allele | | Erroneous  Allele 1 | | Erroneous  Allele 2 | |
| Mixture | mean | STD | mean | STD | | mean | STD | |  | Mixture | mean | STD | mean | STD | mean | STD |
| 0.025% | 0.133% | 0.092% | 0.277% | 0.244% | | 0.204% | 0.173% | |  | 0.025% | 0.032% | 0.012% | 0.011% | 0.012% | 0.009% | 0.004% |
| 0.05% | 0.163% | 0.092% | 0.281% | 0.245% | | 0.212% | 0.178% | |  | 0.05% | 0.055% | 0.011% | 0.010% | 0.010% | 0.008% | 0.004% |
| 0.10% | 0.263% | 0.083% | 0.259% | 0.228% | | 0.193% | 0.166% | |  | 0.10% | 0.167% | 0.019% | 0.012% | 0.012% | 0.009% | 0.005% |
| Pure Control | 0.261% | 0.131% | 0.579% | 0.641% | | 0.531% | 0.397% | |  | Pure Control | 0.011% | 0.016% | 0.011% | 0.009% | 0.013% | 0.019% |
