# Supplementary material for: Detection of Low-Level Mixed-Population Drug Resistance in Mycobacterium tuberculosis Using High Fidelity Amplicon Sequencing
Source: PLoS One. 2015 May 13;10(5):e0126626. doi: 10.1371/journal.pone.0126626 (PMC4430321; doi:10.1371/journal.pone.0126626)
Supplement: S7 Table — (DOCX) [file pone.0126626.s010.docx]

**Table S7. Comparison of MIDGT DST and SMOR analysis**

| Sample ID | INH | | RIF | | AMK | | CAP | | KAN | | MOX | | OFX | |
| --- | --- | --- | --- | --- | --- | --- | --- | --- | --- | --- | --- | --- | --- | --- |
|  | SMOR | DST | SMOR | DST | SMOR | DST | SMOR | DST | SMOR | DST | SMOR | DST | SMOR | DST |
| 2-0112 Pan Sus Control | S | S | S | S | S | S | S | S | S | S | S | S | S | S |
| 21-0017 | R | R | R | R | S | R | S | R | S | R | S | R | S | R |
| 21-0024 | R | R | R | R | S | S | S | S | R | R | R | R | R | R |
| 21-0029 | S | S | S | S | S | S | S | S | S | S | S | S | S | S |
| 21-0031 | R | R | R | R | S | S | S | S | S | S | S | S | S | S |
| 21-0045 | R | R | R | R | S | S | S | S | R | R | S | S | S | S |
| 21-0065 | S | S | S | S | S | S | S | S | S | S | S | S | S | S |
| 21-0067 | R | R | R | R | S | S | S | S | S | S | S | S | S | S |
| 21-0100 | R | R | R | R | S | S | S | S | S | R | S | Flag | S | S |
| 22-0111 | R | R | R | R | S | S | S | S | S | S | S | S | S | S |
| 22-0129 | R | R | R | R | R | R | R | R | R | R | S | S | S | S |
| 23-0070 | R | R | R | R | S | S | S | S | R | R | S | S | S | S |
