# Supplementary material for: Detection of Low-Level Mixed-Population Drug Resistance in Mycobacterium tuberculosis Using High Fidelity Amplicon Sequencing
Source: PLoS One. 2015 May 13;10(5):e0126626. doi: 10.1371/journal.pone.0126626 (PMC4430321; doi:10.1371/journal.pone.0126626)
Supplement: S8 Table — (DOCX) [file pone.0126626.s011.docx]

**Table S8. SMOR results of major population from clinical samples**

| Codon/ promoter | 21-0017 | 21-0024 | 21-0029 | 21-0031 | 21-0045 | 21-0065 | 21-0067 | 21-0100 | 22-0111 | 22-0129 | 23-0070 | Pan Sus Control |
| --- | --- | --- | --- | --- | --- | --- | --- | --- | --- | --- | --- | --- |
| eis -10 | C | T | C | C | C | C | C | C | C | C | C | C |
| eis -12 | G | G | G | G | A | G | G | G | G | G | A | G |
| eis -14 | G | G | G | G | G | G | G | G | G | G | G | G |
| eis -37 | G | G | G | G | G | G | G | T | G | G | G | G |
| eis -43 | T | T | T | T | T | T | T | T | T | T | T | T |
| gyrA 88 | GGC | GGC | GGC | GGC | GGC | GGC | GGC | GGC | GGC | GGC | GGC | GGC |
| gyrA 90 | GCG | GTG | GCG | GCG | GCG | GCG | GCG | GCG | GCG | GCG | GCG | GCG |
| gyrA 91 | TCG | TCG | TCG | TCG | TCG | TCG | TCG | TCG | TCG | TCG | TCG | TCG |
| gyrA 94 | GAC | GAC | GAC | GAC | GAC | GAC | GAC | GAC | GAC | GAC | GAC | GAC |
| gyrA 95 | ACC | ACC | ACC | ACC | ACC | ACC | ACC | ACC | ACC | ACC | ACC | AGC |
| gyrA 112 | GCG | GCG | GCG | GCG | GCG | GCG | GCG | GCG | GCG | GCG | GCG | GCG |
| gyrA 120 | GGC | GGC | GGC | GGC | GGC | GGC | GGC | GGC | GGC | GGC | GGC | GGC |
| gyrA 125 | GCG | GCG | GCG | GCG | GCG | GCG | GCG | GCG | GCG | GCG | GCG | GCG |
| inhA -59 | G | G | G | G | G | G | G | G | G | G | G | G |
| inhA -34 | C | C | C | C | C | C | C | C | C | C | C | C |
| inhA -17 | G | G | G | G | G | G | G | G | G | G | G | G |
| inhA -15 | C | C | C | C | T | C | C | T | T | C | T | C |
| inhA -8 | T | T | T | T | T | T | T | T | T | T | T | T |
| katG 315 | GGT | GGT | GCT | GGT | GGT | GCT | GGT | GGT | GGT | GGT | GGT | GCT |
| rpoB 509 | AGC | AGC | AGC | AGC | AGC | AGC | AGC | AGC | AGC | AGC | AGC | AGC |
| rpoB 511 | CTG | CTG | CTG | CTG | CTG | CTG | CTG | CTG | CTG | CTG | CTG | CTG |
| rpoB 512 | AGC | AGC | AGC | AGC | AGC | AGC | AGC | AGC | AGC | AGC | AGC | AGC |
| rpoB 513 | CAA | CAA | CAA | CAA | CAA | CAA | CAA | CAA | CAA | CAA | CAA | CAA |
| rpoB 515 | ATG | ATG | ATG | ATG | ATG | ATG | ATG | ATG | ATG | ATG | ATG | ATG |
| rpoB 516 | GAC | GAC | GAC | GAC | GAC | GAC | GAC | GTC | GAC | GAC | GAC | GAC |
| rpoB 522 | TCG | TCG | TCG | TCG | TCG | TCG | TCG | TCG | TCG | TCG | TCG | TCG |
| rpoB 526 | CAC | CAC | CAC | CAC | TAC | CAC | TAC | CAC | TAC | CAC | CAC | CAC |
| rpoB 531 | TTG | TTG | TCG | TTG | TCG | TCG | TCG | TCG | TCG | TTG | TTG | TCG |
| rpoB 533 | CTG | CTG | CTG | CTG | CTG | CTG | CTG | CTG | CTG | CTG | CTG | CTG |
| rrs 1401 | A | A | A | A | A | A | A | A | A | G | A | A |
| rrs 1462 | A | A | A | A | A | A | A | A | A | A | A | A |
| rrs 1484 | G | G | G | G | G | G | G | G | G | G | G | G |
| rrs 1486 | A | A | A | A | A | A | A | A | A | A | A | A |
